# Association of plasma proteomics with incident coronary heart disease in individuals with and without type 2 diabetes: results from the population-based KORA study

**DOI:** 10.1186/s12933-024-02143-z

**Published:** 2024-02-03

**Authors:** Hong Luo, Marie-Theres Huemer, Agnese Petrera, Stefanie M. Hauck, Wolfgang Rathmann, Christian Herder, Wolfgang Koenig, Annika Hoyer, Annette Peters, Barbara Thorand

**Affiliations:** 1 Institute of Epidemiology, Helmholtz Zentrum München, German Research Center for Environmental Health (GmbH), Ingolstaedter Landstraße 1, D-85764 Neuherberg, Germany; 2 Institute for Medical Information Processing, Biometry and Epidemiology (IBE), Faculty of Medicine, LMU Munich, Pettenkofer School of Public Health, Munich, Germany; 3 Metabolomics and Proteomics Core, Helmholtz Zentrum München, German Research Center for Environmental Health (GmbH), Neuherberg, Germany; 4 German Center for Diabetes Research (DZD), Partner München-Neuherberg, Neuherberg, Germany; 5 Institute for Biometrics and Epidemiology, German Diabetes Center, Leibniz Center for Diabetes Research at Heinrich Heine Universität, Düsseldorf, Germany; 6 German Center for Diabetes Research (DZD), Partner Düsseldorf, Neuherberg, Germany; 7 Institute for Clinical Diabetology, German Diabetes Center, Leibniz Center for Diabetes Research at Heinrich Heine Universität, Düsseldorf, Germany; 8 Department of Endocrinology and Diabetology, Medical Faculty and University Hospital Düsseldorf, Heinrich Heine Universität, Düsseldorf, Germany; 9 Institute of Epidemiology and Medical Biometry, University of Ulm, Ulm, Germany; 10 Deutsches Herzzentrum München, Technische Universität München, Munich, Germany; 11 German Centre for Cardiovascular Research (DZHK), Partner Site Munich Heart Alliance, Munich, Germany; 12 Biostatistics and Medical Biometry, Medical School OWL, Bielefeld University, Bielefeld, Germany

**Keywords:** Proteomics, Coronary heart disease, Type 2 diabetes, Cohort study, Mendelian randomization

## Abstract

**Background:**

Coronary heart disease (CHD) is a major global health concern, especially among individuals with type 2 diabetes (T2D). Given the crucial role of proteins in various biological processes, this study aimed to elucidate the aetiological role and predictive performance of protein biomarkers on incident CHD in individuals with and without T2D.

**Methods:**

The discovery cohort included 1492 participants from the Cooperative Health Research in the Region of Augsburg (KORA) S4 study with 147 incident CHD cases (45 vs. 102 cases in the group with T2D and without T2D, respectively) during 15.6 years of follow-up. The validation cohort included 888 participants from the KORA-Age1 study with 70 incident CHD cases (19 vs. 51 cases in the group with T2D and without T2D, respectively) during 6.9 years of follow-up. We measured 233 plasma proteins related to cardiovascular disease and inflammation using proximity extension assay technology. Associations of proteins with incident CHD were assessed using Cox regression and Mendelian randomization (MR) analysis. Predictive models were developed using priority-Lasso and were evaluated on top of Framingham risk score variables using the C-index, category-free net reclassification index (cfNRI), and relative integrated discrimination improvement (IDI).

**Results:**

We identified two proteins associated with incident CHD in individuals with and 29 in those without baseline T2D, respectively. Six of these proteins are novel candidates for incident CHD. MR suggested a potential causal role for hepatocyte growth factor in CHD development. The developed four-protein-enriched model for individuals with baseline T2D (ΔC-index: 0.017; cfNRI: 0.253; IDI: 0.051) and the 12-protein-enriched model for individuals without baseline T2D (ΔC-index: 0.054; cfNRI: 0.462; IDI: 0.024) consistently improved CHD prediction in the discovery cohort, while in the validation cohort, significant improvements were only observed for selected performance measures (with T2D: cfNRI: 0.633; without T2D: ΔC-index: 0.038; cfNRI: 0.465).

**Conclusions:**

This study identified novel protein biomarkers associated with incident CHD in individuals with and without T2D and reaffirmed previously reported protein candidates. These findings enhance our understanding of CHD pathophysiology and provide potential targets for prevention and treatment.

**Supplementary Information:**

The online version contains supplementary material available at 10.1186/s12933-024-02143-z.

## Background

Globally, coronary heart disease (CHD) is the leading cause of morbidity and mortality, particularly in Europe, where it accounts for nearly half of all deaths [1]. Although CHD incidence has declined in many countries in recent years, it continues to be a significant public health challenge. Type 2 diabetes (T2D) has been linked to an early onset of CHD and in middle-aged adults the risk of developing CHD is 2–4 times greater in persons with T2D than in those without T2D [2]. Moreover, established classical risk factors for CHD such as blood pressure, serum cholesterol, and smoking are more strongly associated with CHD in persons with diabetes than in those without [3, 4]. Thus, for the effective prevention and management of incident CHD, it is crucial to understand the underlying mechanisms leading to CHD in persons with and without diabetes in the general population.

Advanced proteomics methods such as proximity extension assay (PEA) technology allow the simultaneous measurement of hundreds and even thousands of protein biomarkers [5], which can contribute to the elucidation of unknown biochemical activities and pathways related to disease development and progression. Although several proteomics studies have been conducted for incident CHD [6–10], only a few biomarkers are considered as reliable predictors in clinical practice and treatment guidelines [1, 11] and studies stratifying by diabetes status are lacking. As our and other studies have previously shown, prevalent T2D is strongly associated with various protein biomarkers [12–16]. Furthermore, Elhadad et al. conducted a bidirectional Mendelian randomization (MR) analysis, providing further evidence regarding the influence of T2D on protein levels [13]. Thus, it seems likely that protein–CHD associations could be affected by diabetes status.

Hence, the present study, conducted in the Cooperative Health Research in the Region of Augsburg (KORA) S4 cohort with a 16-year follow-up, explored the potential associations between protein biomarkers and incident CHD separately in individuals with and without T2D. This endeavor aimed to identify both unique and shared pathophysiological pathways and biomarkers potentially involved in the development of CHD in different diabetes states. In addition, we performed MR analysis to further elucidate possible causal effects of the identified biomarkers on incident CHD. Lastly, we evaluated if the identified protein biomarkers improved the predictive performance of incident CHD on top of traditional risk factors for CHD [17]. Our findings were subsequently validated in the prospective KORA-Age1 cohort study among older participants from the general population followed for up to 7.6 years.

## Methods

### Study population

The discovery sample was derived from the population-based KORA S4 cohort study comprising 4261 participants at baseline (1999 to 2001) [18]. The present analysis was confined to individuals aged 55–74 years due to the availability of proteomics data, resulting in a sample of 1653 participants who were followed for a median duration of 15.6 years. After exclusion of participants with missing proteomics data and those with non-T2D (type 1 diabetes and drug-induced diabetes), unclear diabetes status, missing covariables of the main model in the association analysis, prevalent CHD, and those lost to follow-up, a total of 1492 participants remained for analysis (see Supplementary Fig. 1, Additional file 1). Prevalent T2D comprised persons with self-reported and subsequently validated clinically diagnosed T2D and persons with newly diagnosed T2D based on an oral glucose tolerance test (OGTT) using the WHO criteria [19] or baseline glycated hemoglobin (HbA1c) levels ≥ 6.5%. Self-reported T2D was confirmed through questionnaires sent to treating physicians or through medical record reviews. Participants were classified as having clinically diagnosed T2D only if the treating physician reported a diagnosis of T2D, if T2D was documented in the medical records, or if the participants reported taking antidiabetic medication. Finally, the discovery study comprised 228 participants with T2D and 1264 participants without T2D.

For validation, data from the KORA-Age1 study was used. This study includes all participants of the four cross-sectional Monitoring of Trends and Determinants in Cardiovascular Disease (MONICA) Augsburg / KORA surveys conducted in 1984/85 (Survey S1), 1989/90 (Survey S2), 1994/95 (Survey S3), and 1999/2001 (Survey S4), who were born in 1943 or earlier, comprising 9197 participants [20]. Out of these, a sex- and age-stratified random sample of 1079 individuals was extensively examined in 2009 including the collection of blood samples. In the present analysis, data from these participants were used for the validation of the results of the KORA S4 study. After exclusions (see Supplementary Fig. 1, Additional file 1), 888 participants aged 65–93 years who were followed for a median duration of 6.9 years remained for analysis. In the validation study, prevalent T2D was defined based on self-report with subsequent validation as described above, and baseline HbA1c levels ≥ 6.5% only, since no OGTT was conducted in the KORA-Age1 study. Finally, the validation study included 165 participants with T2D and 723 participants without T2D. Out of the 888 participants of KORA-Age1, 206 participants are also part of the KORA S4 discovery sample, since the S4 participants falling into the respective age range were also invited to be part of KORA-Age1 at a later time point as described above.

### Proteomics measurements

At the baseline examinations, venous blood samples were collected while sitting. Plasma samples were stored in liquid nitrogen at − 196 °C until proteomics analysis in 2019–2020 for KORA S4 and in 2023 for KORA-Age1.

The PEA technology by Olink® (Olink Proteomics, Uppsala, Sweden) was used to measure 276 EDTA plasma proteins related to cardiovascular diseases (CVD) and inflammation (CVD-II, CVD-III, and Inflammation panels) in both KORA S4 and KORA-Age1. Detailed measurement procedures were previously outlined [21]. Log2-normalized protein expression values were provided and were normalized by their respective standard deviations within the complete dataset before applying exclusions. Consistent quality control criteria were applied to both the KORA S4 and KORA-Age1 proteomics data. Proteins with over 25% of values below the limit of detection (LOD) were excluded, and proteins measured in duplicate were resolved by retaining the duplicate with fewer LOD values and a lower inter-assay coefficient of variation. Additionally, proteins with missing values were excluded. In the KORA S4 cohort, a total of 233 protein biomarkers were finally included. 76 identified biomarkers associated with incident CHD in the KORA S4 dataset were relevant for the KORA-Age1 validation analysis, and 75 of these biomarkers were included after quality control.

### Outcomes

The combined outcome of CHD encompassed nonfatal myocardial infarction (MI), coronary death, and sudden death, as classified by the International Classification of Disease 9th Revision (410–414 and 798). Until December 2000, the diagnosis of major nonfatal MI was based on the MONICA study algorithm, which considered factors such as symptoms, cardiac enzyme levels (including creatine kinase, aspartate aminotransferase, and lactate dehydrogenase), 12-lead electrocardiograms (ECGs), autopsy results, and history of CHD in fatal cases [22]. From January 2001 onwards, the criteria for diagnosing MI followed the guidelines established by the European Society of Cardiology and the American College of Cardiology [23].

Cases of incident CHD were identified through the KORA Augsburg MI registry, which systematically tracked all fatal and nonfatal MI, in or out of hospital, among residents within the study region aged 25 to 84 years from 2009 onwards [24]. Additionally, regular follow-up questionnaires were administered to the participants. Self-reported incident cases occurring outside the study area and those with self‐reported date of diagnosis falling out of the age range that was covered by the MI registry were further validated using hospital records or by contacting the treating physician. Validation for all coronary deaths was performed through autopsy reports, death certificates, chart reviews or information from the last treating physician. During the study period, KORA S4 study participants underwent two follow-up examinations in 2006–2008 and 2013–2014, which included self-reported information on health status. To further enrich the dataset, postal questionnaires soliciting self-reported health details were dispatched to S4 participants in 2008–2009 and 2016. In contrast, KORA-Age1 participants experienced a singular follow-up examination in 2012 and received postal questionnaires in 2016.

### Baseline measurements / covariates

All participants underwent standard physical and medical examinations at KORA S4 and KORA-Age1 [20, 25]. Trained medical staff conducted interviews to collect information on age, sex, education, smoking habits, alcohol consumption, physical activity, and medical history. Educational attainment was recorded as completed years of schooling. Smoking status was categorized as either current smoker or non-smoker (including never and former smokers). Alcohol intake was categorized into three groups: no consumption (0 g/day), moderate consumption (men: 0.1–39.9 g/day, women: 0.1–19.9 g/day), and high consumption (men: ≥40 g/day, women: ≥20 g/day), based on their self-reported consumption of beer, wine, and liquor on two weekdays and the weekend. Physical activity was assessed as either active or inactive, factoring in the frequency and duration of weekly exercise across different seasons. Medication usage, such as antihypertensive and lipid-lowering drugs, was defined using Anatomical Therapeutic Chemical Classification System codes. Enzymatic methods were used to measure total cholesterol and high-density lipoprotein cholesterol (HDL-cholesterol). Body mass index (BMI) was calculated as weight (kg) divided by height squared (m²). Systolic and diastolic blood pressure were measured on the right arm in a sitting position following the World Health Organization MONICA protocol [26]. Participants without diabetes received a standard 75 g OGTT test in KORA S4. Their blood samples to measure diabetes parameters were taken without stasis after an overnight fast of ≥ 8 h as well as 2 h after the glucose solution ingestion [18].

### Statistical analysis

**Fig. 1:**
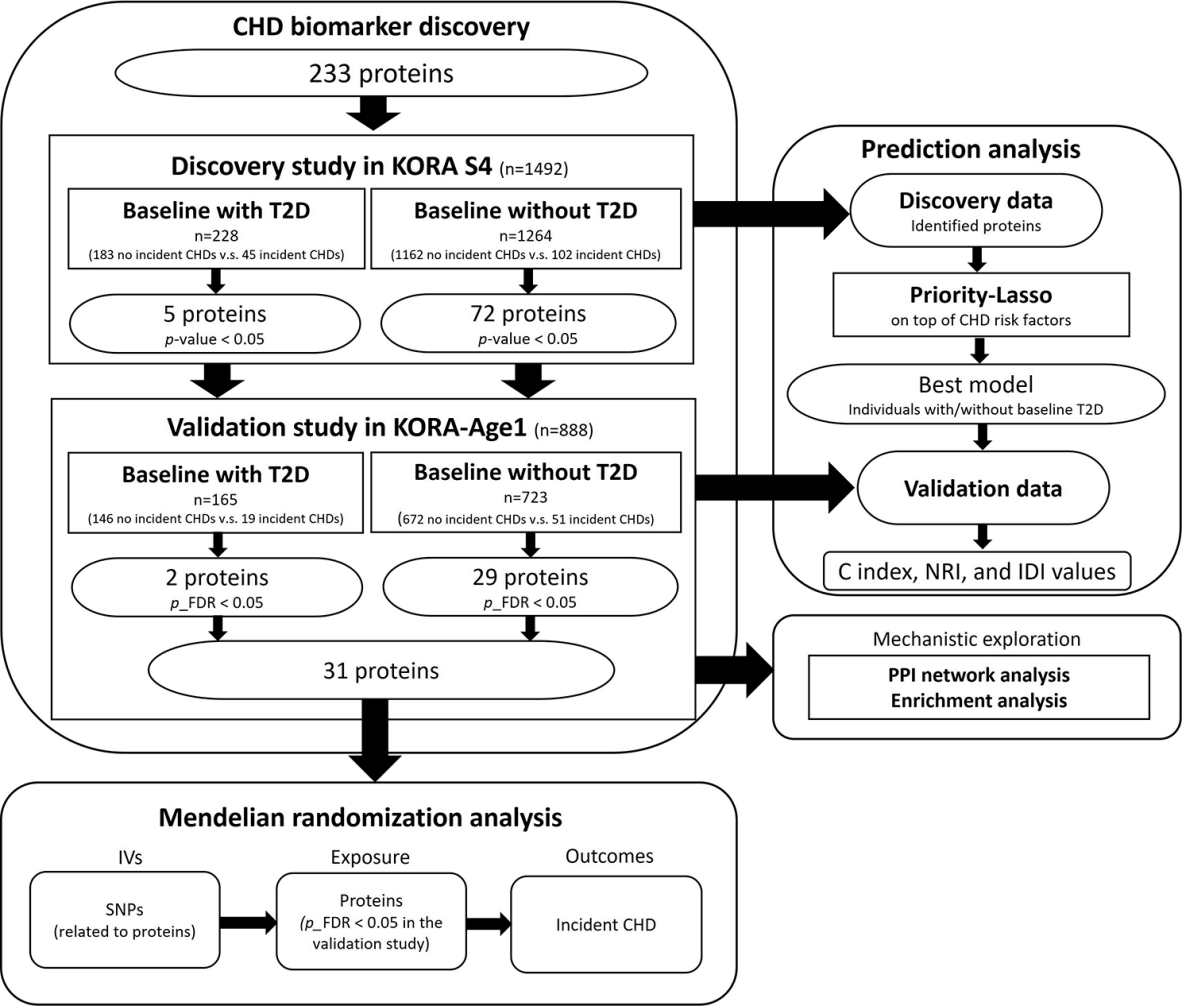
Analysis strategy. CHD: coronary heart disease; C-index: concordance index; FDR: false discovery rate; IDI: integrated discrimination improvement; IV: instrumental variable; NRI: net reclassification index; SNP: single nucleotide polymorphism; T2D: type 2 diabetes

The analysis strategy of the study is shown in Fig. 1.

#### Association analyses of protein biomarkers with coronary heart disease

Associations between each plasma protein level and time to incident CHD were assessed for participants with and without T2D at baseline using Cox proportional hazard regression models. The association analysis was adjusted for important CHD risk factors at baseline: Model 1 (main model) was adjusted for age, sex, total cholesterol, HDL-C, systolic blood pressure, smoking status, and antihypertensive medication usage. These covariates, along with diabetes status, constitute the Framingham Risk Score (FRS) for CHD [17]. Model 2 was further adjusted for additional cardiovascular-related risk factors including BMI, education years, physical activity, alcohol consumption, and lipid-lowering medication usage. Model 3 included fasting status as an independent variable in addition to model 1. The interaction effect of diabetes status was examined by adding diabetes status and the term (protein×diabetes status) to model 1 among the whole KORA S4 participants. Nominally significant (*p*-value < 0.05) proteins in model 1 were validated in the KORA-Age1 study using the same model 1. Proteins with validated significance at the false discovery rate (FDR) lower than 0.05 (*p*_FDR < 0.05), calculated based on the number of nominally significant proteins in KORA S4 (4 for the group with T2D and 71 for the group without T2D), were selected.

To assess whether death as a competing risk influenced the validated associations, we performed a sensitivity analysis using the Fine-Gray subdistribution hazard model to estimate the CHD incidence over time in the presence of death as a competing risk. In another sensitivity analysis, we excluded the overlapping participants, who participated in both the KORA S4 and KORA-Age1 study, from the KORA-Age1 analysis (*n* = 37 in the group with T2D and *n* = 169 in the group without T2D).

#### Two-sample mendelian randomization analysis

A two-sample MR was applied using the published available genome-wide association study (GWAS) data of European ancestry to explore the potential causal links between biomarkers and incident CHD. For the validated protein biomarkers, the instrumental variables (IVs) were extracted from the Olink-based GWAS database, which included 54,219 participants from the UK biobank [27]. Incident CHD GWAS data were from Mbatchou J et al., involving 352,063 participants from the UK Biobank dataset (case-control ratio = 1:11) [28]. The IV selection involved identifying SNPs associated with proteins at a significance threshold of *p-*value < 5 × 10^− 8^, focusing on *cis* regions, and eliminating ambiguous palindromic SNPs with A/T or G/C alleles. To test the assumption of MR, SNP independence was verified via PhenoScanner V2 database (http://www.phenoscanner.medschl.cam.ac.uk/), and SNPs associated with traditional CHD risk factors were removed. The remaining SNPs were clumped using an r^2^ = 0.001 threshold to eliminate the linkage disequilibrium with the lead SNP. SNPs were then extracted as IVs from the outcome’s GWAS.

For causal assessment, the Wald ratio test was performed when only one IV was available, and the inverse variance-weighted method was used for proteins with at least two IVs [29, 30]. The significant threshold was adjusted using the Bonferroni correction (*p-*value = 0.05 divided by the number of tested proteins). Sensitivity analyses evaluated instrument heterogeneity and directional horizontal pleiotropy using Cochran’s Q test and MR-Egger regression.

#### Network analysis and enrichment analysis

To elucidate potential connections and mechanisms of the selected proteins, we annotated the above validated two proteins in the group with T2D and 29 proteins in the group without T2D, respectively, using the STRING database version 12.0 (https://string-db.org/). Based on the built network of identified proteins, enrichment analysis was performed to detect pathways linked to CHD based on the Reactome pathway knowledgebase [31]. Given the limited pool of identified proteins (*n* = 2) in the group with T2D, the enrichment analysis was performed only for the 29 proteins identified in the group without T2D.

#### Prediction of incident coronary heart disease

KORA S4 served as the discovery dataset, while KORA-Age1 was used as the validation dataset in the prediction analysis. The components of the FRS (model 1 in the association analyses) were used as the basic model for CHD prediction.

To enhance the accuracy and effectiveness of constructing a predictive model, only the protein biomarkers significantly associated with incident CHD in the discovery analysis were included in the predictor selection for the extended model (basic model + protein biomarkers) using the priority-Lasso which is a least absolute shrinkage and selection operator (LASSO)-based intuitive analysis strategy and constructs a prediction model for a clinical outcome by defining the blocks of different types of predictor variables [32]. There were five and 72 proteins selected for participants with T2D and without T2D in KORA S4, respectively, of which one in both groups (melusin [ITGB1BP2]) failed quality control in KORA-Age1 and was therefore excluded from further analyses. The penalization parameter λ was determined by five-fold cross-validation with Cox regression design. In the discovery dataset, we fixed the seven FRS variables as block 1 to prevent any shrinkage by priority-Lasso, while the identified proteins (4 / 71 proteins) were incorporated as block 2 for their respective T2D status groups. The performance of the priority-Lasso protein-extended model was compared to the basic model in both the KORA S4 dataset and the KORA-Age1 dataset. The performance of the basic and extended model was evaluated through three measures: (1) Harrel’s concordance index (C-index) for the basic model, the protein-extended model, and their difference (ΔC‐index = C‐index _extended_ - C‐index _basic_) [33]; (2) the category-free net reclassification index for all participants combined (cfNRI), for incident CHD cases (cfNRI_cases_), and for non-CHD controls (cfNRI_controls_) [34]; (3) the absolute integrated discrimination improvement (IDI) [35]. All effect estimates were calculated as the arithmetic mean of these measures using five-fold cross-validation. Their corresponding confidence intervals were calculated using 100x bootstrapping.

The R version 4.3 (https://www.r-project.org/) was used for all analyses.

## Results

### Baseline characteristics of the study participants

Table 1 presents the characteristics of the study participants at baseline. In the KORA S4 study, 45 and 102 participants had incident CHD in the group with and without T2D at baseline (15.5 vs. 5.6 per 1000 person-years), respectively. In the KORA-Age1 study, 19 and 51 participants had incident CHD in the group with and without T2D at baseline (18.7 vs. 10.7 per 1000 person-years), respectively.

**Table 1:** Baseline characteristics of the study population

Characteristics	Discovery study - KORA S4				Validation study - KORA-Age1			
	With T2D (*n* = 228)		Without T2D (*n* = 1264)		With T2D (*n* = 165)		Without T2D (*n* = 723)	
	CHD cases (*n* = 45)	Noncases (*n* = 183)	CHD cases (*n* = 102)	Noncases (*n* = 1162)	CHD cases (*n* = 19)	Noncases (*n* = 146)	CHD cases (*n* = 51)	Noncases (*n* = 672)
Age (years)	66 (62, 70)	64 (60, 69)	67 (61, 70)	63 (59, 68)	79 (74, 85)	76 (71, 80)	77 (73, 81)	74 (69, 80)
Male (%)	35 (77.8)	91 (49.7)	68 (66.7)	546 (47.0)	8 (42.1)	73 (50.0)	32 (62.7)	307 (45.7)
Systolic blood pressure (mmHg)	138.5 (130.5, 161.0)	145.0 (132.5, 156.0)	141.0 (128.1, 151.5)	132.5 (120.0, 146.0)	146.0 (132.0, 169.8)	137.8 (124.0, 149.4)	139.5 (126.0, 152.3)	137.0 (124.5, 149.5)
Diastolic blood pressure (mmHg)	80.0 (73.5, 91.0)	82.5 (76.0, 88.5)	80.8 (74.0, 87.5)	79.5 (73.0, 86.5)	75.0 (63.3, 85.8)	73.5 (66.5, 82.0)	73.0 (68.0, 83.3)	76.5 (70.0, 83.0)
Total cholesterol (mmol/l)	5.8 (5.3, 6.6)	6.0 (5.3, 6.8)	6.2 (5.7, 6.9)	6.3 (5.6, 7.0)	4.9 (4.4, 5.3)	5.2 (4.5, 5.9)	5.2 (4.3, 6.3)	5.6 (4.9, 6.3)
HDL-cholesterol (mmol/l)	1.2 (1.0, 1.4)	1.3 (1.1, 1.5)	1.4 (1.1, 1.7)	1.5 (1.3, 1.8)	1.3 (1.1, 1.6)	1.2 (1.1, 1.5)	1.2 (1.0, 1.6)	1.5 (1.2, 1.7)
BMI (kg/m^2^)	30.6 (28.1, 34.3)	30.2 (27.6, 33.4)	28.4 (26.2, 30.7)	27.6 (25.3, 30.4)	29.4 (25.7, 33.0)	30.6 (27.9, 34.4)	27.3 (25.3, 30.1)	27.6 (25.3, 30.2)
Fasting glucose (mmol/l) ^a^	7.5 (6.5, 8.3)	7.0 (6.2, 7.6)	5.5 (5.1, 5.9)	5.4 (5.1, 5.8)	-	-	-	-
2-hour glucose (mmol/l) ^b^	12.3 (9.8, 14.9)	12.1 (10.4, 13.5)	6.8 (5.8, 8.0)	6.2 (5.1, 7.3)	-	-	-	-
Fasting insulin (pmol/l) ^c^	78.8 (47.9, 99.9)	81.0 (53.8, 129.4)	69.3 (47.7, 99.9)	57.6 (40.5, 81.0)	-	-	-	-
HbA1c (mmol/mol) ^d^	50.0 (43.0, 66.0)	45.0 (40.0, 52.0)	38.0 (34.0, 40.0)	38.0 (36.0, 40.0)	48.6 (44.0, 55.2)	47.5 (42.3, 51.7)	37.7 (33.7, 39.4)	36.6 (34.4, 39.0)
HbA1c (%) ^d^	6.7 (6.1, 8.2)	6.3 (5.8, 6.9)	5.6 (5.3, 5.8)	5.6 (5.4, 5.8)	6.6 (6.2, 7.2)	6.5 (6.0, 6.9)	5.6 (5.2, 5.8)	5.5 (5.3, 5.7)
Fasting status (yes, %)	23 (51.1)	113 (61.7)	98 (96.1)	1083 (93.2)	0 (0)	8 (5.5)	4 (7.8)	44 (6.5)
Education (years)	10 (8, 11)	10 (8, 11)	10 (10, 12)	10 (10, 12)	10 (9, 10)	10 (10, 11)	10 (10, 12)	10 (10, 12)
Physical activity (active, %) ^e^	5 (11.1)	60 (33.0)	36 (35.6)	524 (45.1)	7 (36.8)	80 (54.8)	21 (41.2)	398 (59.2)
Current smoker (%)	10 (22.2)	24 (13.1)	17 (16.7)	157 (13.5)	1 (5.3)	6 (4.1)	4 (7.8)	27 (4.0)
Alcohol consumption (%) ^e^								
None	16 (35.6)	64 (35.2)	26 (25.7)	306 (26.4)	11 (57.9)	61 (41.8)	19 (37.3)	223 (33.2)
Moderate	20 (44.4)	86 (47.2)	55 (54.5)	611 (52.6)	8 (42.1)	69 (47.3)	19 (37.3)	345 (51.3)
High	9 (20.0)	32 (17.6)	20 (19.8)	244 (21.0)	0 (0)	16 (10.9)	13 (25.4)	104 (15.5)
Medication use (%)								
Antihypertensive drugs	20 (44.4)	94 (51.4)	49 (48.0)	361 (31.1)	18 (94.7)	122 (83.6)	38 (74.5)	431 (64.1)
Statins	4 (8.9)	27 (14.8)	9 (8.8)	91 (7.8)	8 (42.1)	52 (35.6)	14 (27.5)	149 (22.2)
Lipid-lowering drugs	6 (13.3)	32 (17.5)	11 (10.8)	107 (9.2)	8 (42.1)	56 (38.4)	15 (29.4)	153 (22.8)

Note: Data are presented as median (25th, 75th percentile) for continuous variables and n (%) for categorical variables

Abbreviations: BMI: body mass index; CHD: coronary heart disease; HbA1c: haemoglobin A1c; HDL: high-density lipoprotein; T2D: type 2 diabetes

^a^ Data were calculated in 129 participants with T2D (20 CHD cases vs. 109 noncases) and 1173 participants without T2D (98 CHD cases vs. 1075 noncases) at KORA S4.

^b^ Data were calculated in 121 participants with T2D (18 CHD cases vs. 103 noncases) and 1141 participants without T2D (96 CHD cases vs. 1045 noncases) at KORA S4.

^c^ Data were calculated in 134 participants with T2D (22 CHD cases vs. 112 noncases) and 1141 participants without T2D (95 CHD cases vs. 1046 noncases) at KORA S4.

^d^ Data were calculated in 1262 participants without T2D (102 CHD cases vs. 1160 noncases) at KORA S4.

^e^ Data were calculated in 227 participants with T2D (45 CHD cases vs. 182 noncases) and 1261 participants without T2D (101 CHD cases vs. 1161 noncases) at KORA S4

### Associations of protein biomarkers with coronary heart disease

**Fig. 2:**
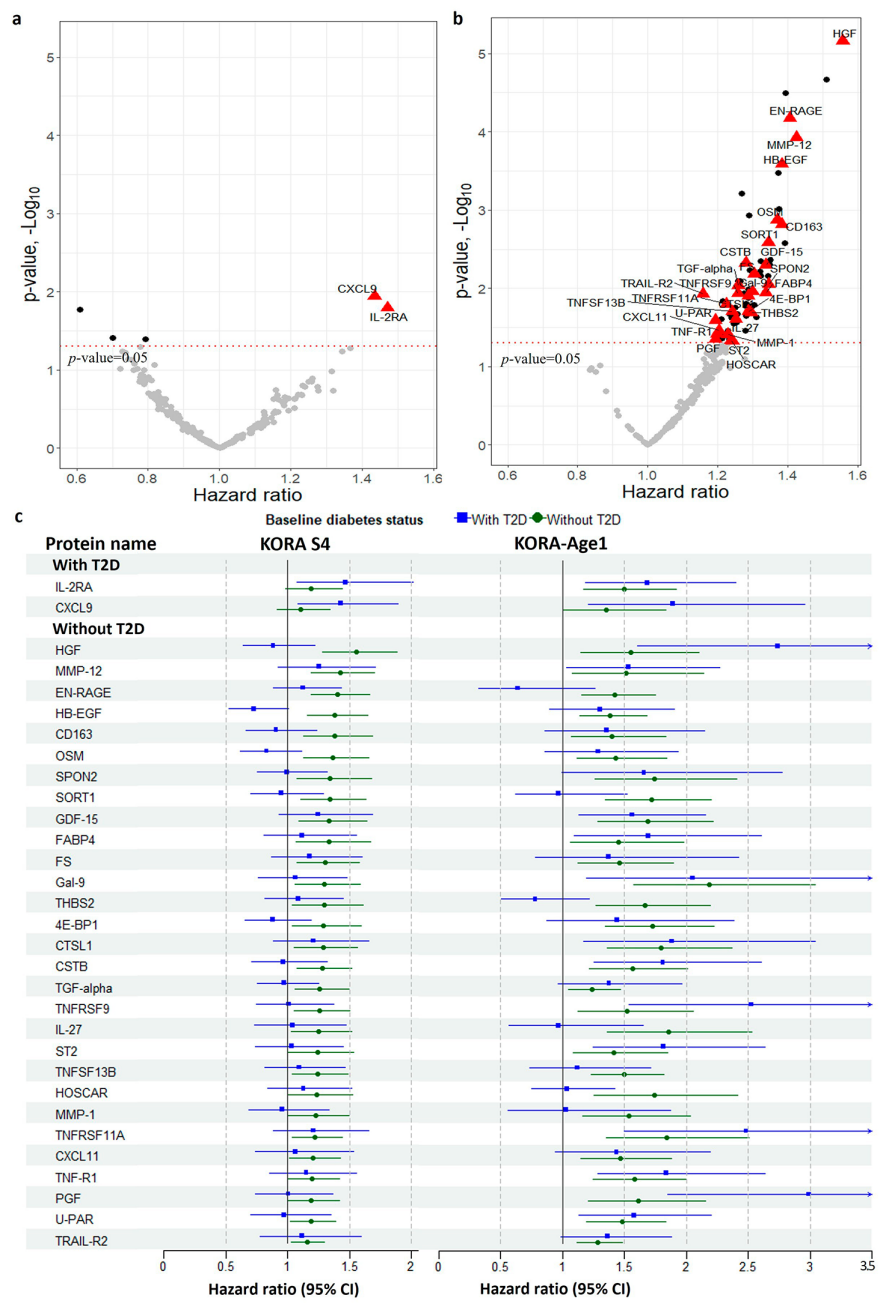
Association of 233 proteins with incident coronary heart disease in individuals **(a)** with type 2 diabetes (T2D), and **(b)** without T2D at baseline. Hazard ratios have been calculated per 1 SD increase in normalized protein expression values on a log2 scale. Effect estimates and *p-*values were derived from Cox regression analysis adjusted for age, sex, total cholesterol, high-density lipoprotein cholesterol, systolic blood pressure, antihypertensive medication use, and current smoking (Model 1). The red triangles represent the validated proteins in the validation study, identified using the false discovery rate (*p*_FDR < 0.05). The black dots represent significant proteins at the uncorrected level (*p* < 0.05) in the discovery study which were not replicated in the validation study. **(c)** Forest plot of validated proteins in KORA S4 and KORA-Age1 cohorts stratified by T2D status

In the KORA S4 study, five protein biomarkers showed nominally significant associations with incident CHD in the group with T2D, whereas a total of 72 biomarkers were significant in the group without T2D in model 1 (see Supplementary Table 1, Additional file 2). ITGB1BP2 failed the quality control in KORA-Age1 and was consequently excluded from the validation study. Of the remaining 4 and 71 biomarkers, two and 29 protein biomarkers, respectively, were successfully validated in the KORA-Age1 dataset after correction for multiple testing (see Fig. 2 and Supplementary Table 2, Additional file 2). The correlations between these 31 validated protein biomarkers are illustrated in Supplementary Fig. 2, Additional file 1. Notably, there was no overlap in significant proteins between the two distinct diabetes status groups in the validation study.

After further adjusting for other lifestyle factors in model 2, five of the validated proteins (osteoclast-associated immunoglobulin-like receptor [HOSCAR], placenta growth factor [PGF], thrombospondin-2 [THBS2], ST2 protein, and tumor necrosis factor receptor 1 [TNF-R1]) lost significance in the group without T2D. After further accounting for baseline fasting status in model 3, all associations from model 1 remained significant (see Supplementary Table 1, Additional file 2). In the KORA S4 study population which was used as the discovery study, 51 proteins showed significant interactions with T2D status supporting our hypothesis that associations of proteins with CHD may be modified by the presence of T2D. Eight out of the 33 validated proteins displayed a significant interaction effect with diabetes status in the discovery study (see Supplementary Table 1, Additional file 2), but none of these interaction effects were validated in the KORA-Age1 study.

Considering death as a competing risk, two of the validated proteins (tumor necrosis factor receptor superfamily member 9 [TNFRSF9], and fatty acid-binding protein 4 [FABP4]) were no longer significantly associated with incident CHD in both cohorts in those without T2D (see Supplementary Table 3, Additional file 2). After excluding overlapping KORA S4 participants from the KORA-Age1 sample, eight proteins (C-X-C motif chemokine 9 [CXCL9], interleukin-2 receptor subunit alpha [IL-2RA], follistatin [FS], matrix metalloproteinase-12 [MMP-12], hepatocyte growth factor [HGF], oncostatin-M [OSM], TNFRSF9, and scavenger receptor cysteine-rich type 1 protein M130 [CD163]) lost significance after multiple testing regarding their association with incident CHD (see Supplementary Table 4, Additional file 2).

### Causal effects of validated proteins on coronary heart disease

**Fig. 3:**
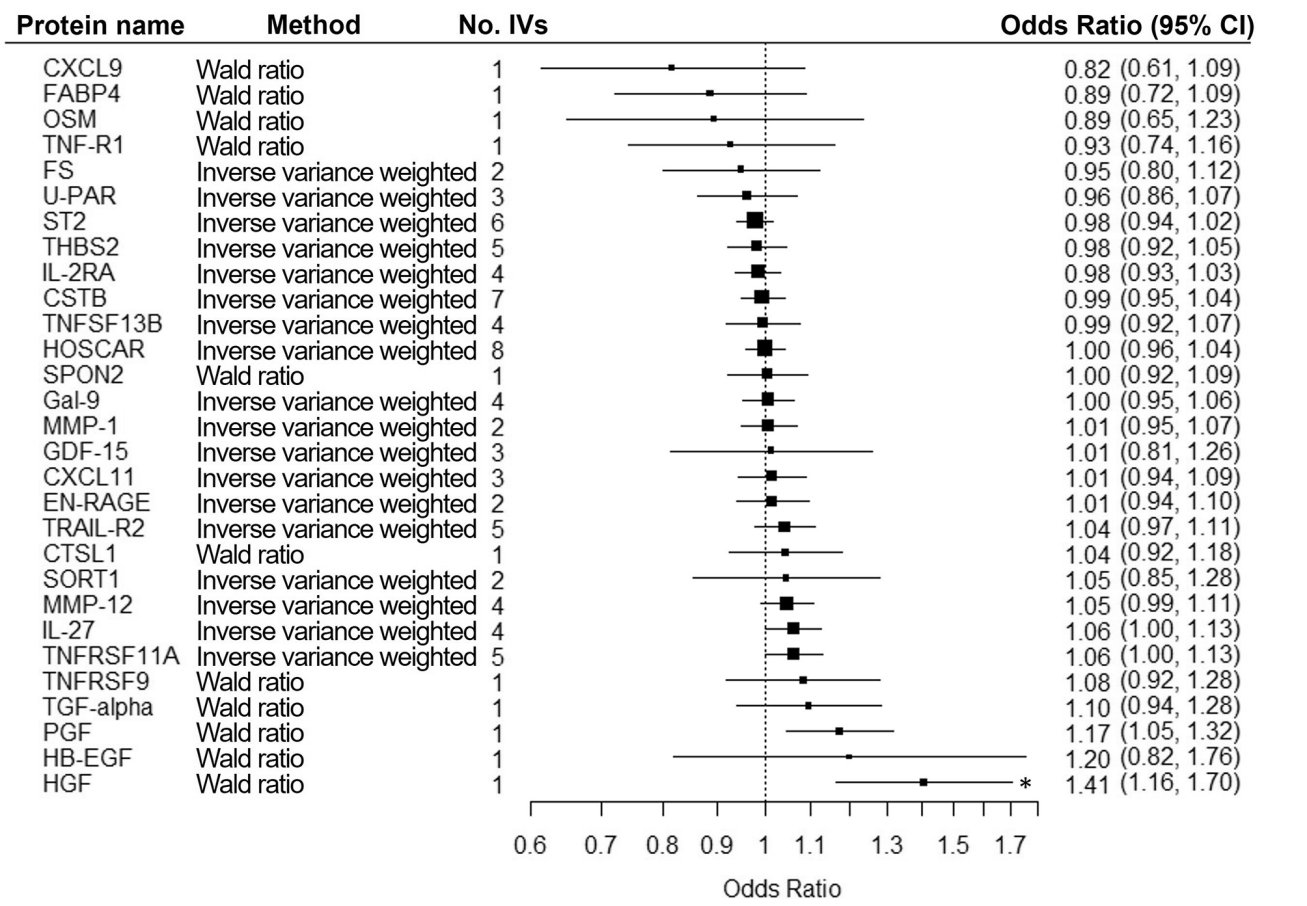
Mendelian Randomization analysis results of the validated proteins as exposure and coronary heart disease as outcome.* The protein remained significant after correction for multiple testing using the Bonferroni method (*p*_significant_ = 0.05/29 = 0.00172)

Cis-acting genetic IVs were identified for 29 validated CHD-associated proteins from previous GWAS data and their potential causal effects were assessed (see Fig. 3 and Supplementary Table 5, Additional file 2). Two proteins (eukaryotic translation initiation factor 4E-binding protein 1 [4E-BP1] and CD163) lacked qualified IVs. HGF was the only protein with a statistically significant causal effect on CHD after correction for multiple testing (Wald ratio, b = 0.3422; *p*-value = 0.0004), while PGF lost significance following correction for multiple testing (Wald ratio, b = 0.1607; *p*-value = 0.0068).

### Network and related pathways of identified proteins

**Fig. 4:**
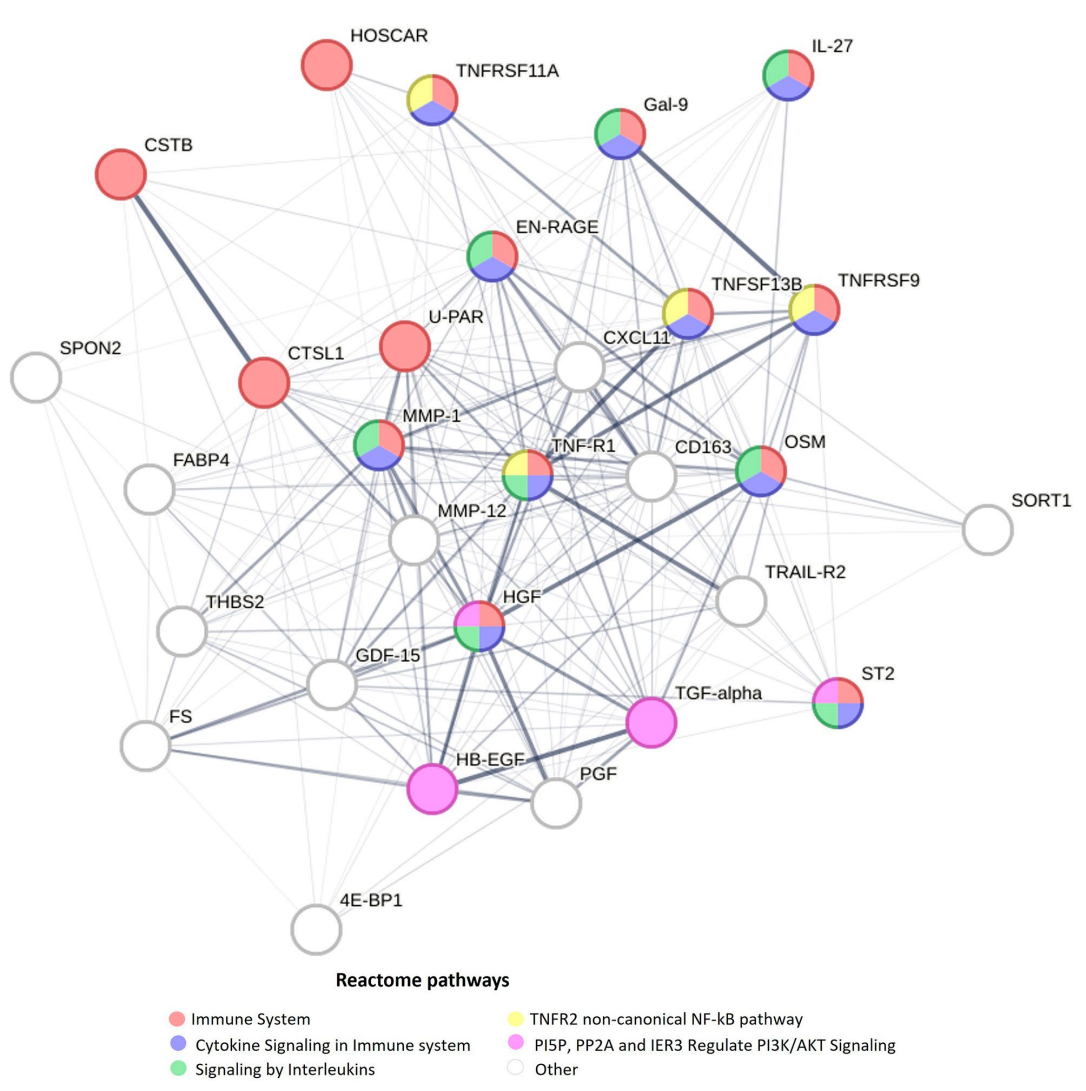
Protein-protein interaction network of validated coronary heart disease-associated proteins among participants without type 2 diabetes at baseline. The edges between protein nodes represent the interaction score between the proteins from the STRING database considering all types of evidence. Only edges featuring interaction scores > 0.15 are displayed. The thickness of edges corresponds to the strength of data support. Node color signifies the Reactome pathway the protein is associated with. The 5 most enriched Reactome pathways are displayed

To unravel biological insights, network analysis and pathway analysis were performed on the 29 validated proteins in the group without T2D at baseline. The following Reactome pathways (number of involved proteins) were overrepresented in the protein biomarker set: immune system (*n* = 15), cytokine signaling in immune system (*n* = 11), signaling by interleukins (*n* = 8), and TNFR2 non-canonical NF-kB pathway (*n* = 4) and PI5P, PP2A and IER3 regulate PI3K/AKT signaling (*n* = 4). The resulting protein-protein interaction network is visualized in Fig. 4. TNF-R1 was involved in the top four pathways and emerged as a central player within the network.

### Prediction of incident coronary heart disease

In the group with T2D, the four identified proteins (carboxypeptidase A1 [CPA1], IL-2RA, CXCL9, and neurotrophin-3 [NT-3]) were all selected by priority-Lasso on top of the basic model. While the basic model yielded a C-index of 0.693 (95% CI = 0.563–0.774), the protein-extended model showed improved predictive performances with ΔC-index of 0.017 (95% CI = 0.006–0.145), cfNRI of 0.253 (95% CI = 0.024–0.497) and IDI of 0.051 (95% CI = 0.014–0.124) in the KORA S4 sample. In the KORA-Age1 sample, the protein-extended model improved only the cfNRI with a value of 0.633 (95% CI = 0.139–1.075) which was mainly driven by an increased cfNRI_controls_ (0.506, 95% CI = 0.288–0.796).

In the group without T2D, 12 proteins (CD163, epithelial cell adhesion molecule [Ep-CAM], osteopontin [OPN], TNF-R1, kidney injury molecule 1 [KIM1], proheparin-binding EGF-like growth factor [HB-EGF], MMP-12, protein-glutamine gamma-glutamyltransferase 2 [TGM2], vascular endothelial growth factor A [VEGF-A], interleukin-6 [IL-6], IL-10, and protein S100-A12 [EN-RAGE]) were selected alongside the basic FRS variables in the priority-Lasso analysis. The basic model yielded a C-index of 0.700 (95% CI = 0.657–0.760) in the KORA S4 sample and 0.683 (95% CI = 0.564–0.769) in the KORA-Age1 sample, respectively. This augmented model led to improved predictive performances in both the KORA S4 and KORA-Age1 datasets, yielding enhanced ΔC-index (0.038, 95% CI = 0.024–0.133) and overall cfNRI (0.465, 95% CI = 0.027–0.741) in the KORA-Age1 sample which was mainly driven by an increased cfNRI_controls_ (0.380, 95% CI = 0.273–0.533) as depicted in Table 2. The estimates of the extended model for incident CHD in KORA S4 are presented in Supplementary Tables 6–7, Additional file 2.

In the sensitivity analysis excluding 206 overlapping participants from the KORA-Age1 sample, prediction results were very similar. In the group with T2D, the established model showed improved predictive performances based on cfNRI (0.522 [95% CI = 0.363–0.925]) which was mainly driven by the cfNRI_controls_ (0.488 [95% CI = 0.329–0.625]), while in the group without T2D, an enhanced ΔC-index of 0.048 (95% CI = 0.029–0.130) and an overall cfNRI of 0.262 (95% CI = 0.003–0.760) were observed (see Supplementary Table 8, Additional file 2).

**Table 2:** Predictive performance of selected protein biomarkers for incident coronary heart disease on top of framingham risk score (FRS) components

Baseline status	KORA S4		KORA-Age1	
	Basic model	Extended model	Basic model	Extended model
**With T2D** ^**a**^				
C-index	0.693 [0.563; 0.774]	0.711 [0.656; 0.810]	0.642 [0.545; 0.861]	0.667 [0.652; 0.876]
ΔC-index	-	0.017 [0.006; 0.145]	-	0.025 [-0.037; 0.142]
cfNRI	-	0.253 [0.024; 0.497]	-	0.633 [0.139; 1.075]
cfNRI_cases_	-	0.038 [-0.022; 0.401]	-	0.127 [-0.373; 0.493]
cfNRI_controls_	-	0.215 [0.116; 0.465]	-	0.506 [0.288; 0.796]
IDI	-	0.051 [0.014; 0.124]	-	0.017 [-0.045; 0.174]
**Without T2D** ^**b**^				
C-index	0.700 [0.657; 0.760]	0.754 [0.729; 0.817]	0.683 [0.564; 0.769]	0.721 [0.627; 0.812]
ΔC-index	-	0.054 [0.026; 0.102]	-	0.038 [0.024; 0.133]
cfNRI	-	0.462 [0.325; 0.742]	-	0.465 [0.027; 0.741]
cfNRI_cases_	-	0.087 [-0.002; 0.321]	-	0.085 [-0.312; 0.268]
cfNRI_controls_	-	0.375 [0.292; 0.462]	-	0.380 [0.273; 0.533]
IDI	-	0.024 [0.020; 0.076]	-	0.006 [-0.005; 0.076]

Abbreviations: C-index: concordance Index; cfNRI: category‐free net reclassification index; IDI: independent discrimination improvement; T2D: type 2 diabetes

^a^ Basic model (FRS components): age, sex, total cholesterol, HDL-cholesterol, systolic blood pressure, antihypertensive medication use, and current smoking. Extended model: Basic model + CPA1 + IL2RA + CXCL9 + NT-3

^b^ Basic model (FRS components): age, sex, total cholesterol, HDL-cholesterol, systolic blood pressure, antihypertensive medication use, and current smoking. Extended model: Basic model + CD163 + Ep-CAM + OPN + TNF-R1 + KIM1 + HB-EGF + MMP-12 + TGM2 + VEGF-A + IL-6 + IL-10 + EN-RAGE

## Discussion

We conducted a longitudinal analysis to investigate the proteomic profile of incident CHD among individuals with different baseline diabetes status. Only two validated proteins were identified for incident CHD in individuals with T2D, while twenty-nine validated proteins were identified in those without T2D, respectively. Among the 31 proteins, six proteins (TNFRSF13B, THBS2, transforming growth factor-alpha [TGF-alpha]), CXCL9, CXCL11, and 4E-BP1) are novel candidate biomarkers for CHD. Additionally, the two-sample MR approach provided suggestive evidence for a causal effect of HGF on CHD.

### Novel protein biomarkers associated with CHD

Several novel incident CHD-related protein biomarkers identified in the present study have previously been demonstrated to be related to atherosclerosis and CHD progression. Among these, increased serum levels of TNFRSF13B, a TNF superfamily receptor, have been linked to the presence of plaque, i.e. subclinical CHD [36]. Furthermore, we identified THBS2 as a marker of incident CHD, which is a matricellular protein facilitating cell-matrix interactions, that was positively associated with both incident heart failure (HF) hospitalization and deterioration in diastolic function in a recent study [37]. TGF-alpha, which directly activates the transcription factor NF-κB through the epidermal growth factor receptor pathway, was previously found to be associated with higher cardiovascular mortality in patients with chronic CHD [38].

Other novel biomarkers identified by our study include CXCL9 and CXCL11, which are inflammatory chemokines known to induce immune cell infiltration through the C-X-C motif chemokine receptor 3 (CXCR3). Previous investigations have suggested the involvement of CXCLs and CXC receptors in distal sensorimotor polyneuropathy, various CVDs as well as T2D [39–41]. This may partly elucidate the specific role of CXCL9, particularly in individuals with baseline T2D in the context of incident CHD. Furthermore, we found a positive association between 4E-BP1 and incident CHD, which is a substrate of the mTOR-containing multiprotein complex-1 (mTORC1) with the capacity to inhibit translation initiation. This protein has been assumed to play a crucial role in regulating the viability of cardiomyocytes, particularly in the context of heart failure [42]. This novel set of protein biomarkers presents new avenues for exploring potential prevention strategies and therapeutic targets addressing CHD.

### Confirmed protein biomarkers associated with CHD

Our validated CHD-related biomarkers align with previous investigations of proteomic biomarkers using the same Olink panels to identify associations with incident CHD [6–9, 43, 44]. It is noteworthy that the aforementioned studies predominantly assessed associations of proteins with incident CHD in population-based samples comprising participants with and without diabetes together, whereas our study stands out as the first to identify biomarkers specifically according to baseline T2D status. Growth differentiation factor-15 (GDF-15), a member of the transforming growth factor-β cytokine superfamily, is known to severely increase during oxidative stress and inflammation, which suggests GDF-15 as a credible marker for the increased risk of incident CHD [6, 9]. TNF-related apoptosis inducing receptor 2, a TNF superfamily member, has been associated with a higher risk of incident MI, possibly due to its role in inflammation and apoptosis [6]. One of the metalloproteases involved in the breakdown of collagen and elastin, MMP-12, has also been positively associated with both incident MI and HF [6, 9]. Another of the identified proteins that plays a role in inflammation is urokinase plasminogen activator surface receptor, which is closely linked with immune and inflammatory activation and was associated with an increasing risk of incident MI [6]. FABP4, which is secreted by adipocytes, has well-documented implications for insulin resistance and atherosclerosis, consistently showing elevated levels in persons developing incident CHD [7]. Similar results in studies on incident CHD and cardiovascular mortality have been reported for CD163, a marker involved in macrophage activation [7, 8]. Additionally, FS is secreted from the liver and was reported to be associated with a higher risk of incident coronary events, independently of established risk factors including diabetes, using PEA technology [43]. Cystatin B, an inhibitor of cathepsin L, was associated with an elevated risk of incident CHD in the highest tertile [44].

Similar findings for incident CHD were observed regarding HGF, TNF-R1, PGF, EN-RAGE, and IL-2RA when proteins were measured using other methods, such as ELISAs. HGF in particular, emerges as a pivotal protein known for its effects on CVD, activating pathways that counteract apoptosis, inflammation, oxidation, and fibrosis [45]. Our MR analysis revealed a suggestive positive causal association of HGF on incident CHD in the general population, with a consistent directionality with our findings of association analysis in individuals without baseline T2D. Previous study supported our observational and MR analysis findings [46]. Of note, this is the first study that provides suggestive evidence for a causal association between HGF and incident CHD. TNF-R1, a crucial proinflammatory cytokine mediator, was associated with an increased risk of incident CHD, especially in women [47]. PGF, a VEGF homologue and EN-RAGE, an endogenously produced inflammatory ligand, were associated with a higher risk of incident CHD [48, 49]. Moreover, serum IL-2RA, a marker of T lymphocyte activation, was significantly positively associated with incident CHD in participants with T2D in the present study. In line with our findings, an increased risk for incident CHD as well as prevalent T2D was reported in older adults, but the effect of IL-2RA was not specifically tested on incident CHD in persons with baseline T2D [50].

In line with our findings, cathepsin L1, gal-9, spondin-2, and TNFRSF11A measured using PEA technology were significantly altered in patients with prevalent CHD compared to participants without CHD [51]. Additionally, other proteins including HB-EGF, IL-27, sortilin, matrix metalloproteinase-1, OSM, ST2, TNFRSF9, and HOSCAR were reported to show higher concentrations in blood samples from individuals with CHD compared with healthy controls or non-CHD participants [52–59].

Our pathway and network analysis for the 29-incident CHD-related protein biomarkers among individuals without baseline T2D revealed insights into the mechanistic underpinnings of CHD pathogenesis. Notably, the enrichment of pathways such as the immune system, cytokine signaling in the immune system, signaling by interleukins, and the TNFR2 non-canonical NF-kB pathway underscored the role of inflammatory processes in CHD development. This aligned with established literature highlighting the significance of immune response and cytokine signaling in the development of CHD [60, 61]. Importantly, TNF-R1, a central player in our identified pathways, has been implicated in mediating inflammatory responses, reinforcing its potential key role in the pathogenesis of incident CHD. Our findings exhibited substantial overlap with previously identified pathways [60, 61], providing further support for the involvement of inflammatory mechanisms in CHD development.

### Prediction of CHD through protein biomarkers

Our study is the first to establish proteomics-enriched predictive models for incident CHD separately for those with and without prevalent T2D. However, it is noteworthy that in the validation study, protein-enriched models significantly improved the predictive performance based on selected performance measures only, particularly among those with T2D. Among participants without T2D, the model enriched with 12 proteins improved discrimination of incident CHD by 5.6% based on the C-index (delta C-index = 0.038) compared with traditional CHD risk factors in the validation study. Our findings partly coincide with those of Lind et al., who utilized the Olink CVD I panels to derive a 7-protein enriched model for the prediction of the 15-year risk of incident CVD (including MI, ischemic stroke and HF) [6] in a population including about 11% of persons with prevalent diabetes. This approach resulted in a 7.3% improvement compared with traditional risk factors in the replication sample. Hereby, EN-RAGE was the only biomarker that overlapped with our selected proteins. Similarly, McCarthy et al. established a protein model measured using the Luminex xMAP platform and reported a 3.6% improvement in predicting incident major adverse cardiovascular events (including cardiovascular death, MI, and stroke) during a 3.6-year follow-up period [62]. However, the application of these models in clinical practice needs careful consideration, given the differences in biomarkers, populations, and methodologies across studies.

### Study strengths and limitations

We used advanced targeted proteomics technology to examine a wide range of proteins linked to CHD. A major strength of the statistical analysis constitutes the validation of the identified proteins in another cohort study. Specifically exploring protein-CHD associations by diabetes status provided evidence of the underlying mechanisms leading to CHD in persons with and without T2D. By analyzing genetic data using a Mendelian randomization approach, we gained insights into potential causal relationships between proteins and CHD risk.

However, there are some limitations to consider. First, due to the limited number of incident CHD cases, our analyses may not be sufficiently powered to detect a difference in CHD vs. no CHD groups, particularly in the group with T2D at baseline. Along these lines, due to the limited number of validated proteins in those with T2D, pathway analyses had to be restricted to those without T2D. Adjusting for multiple testing was necessary due to the numerous analyses, but could have caused overcorrection. Additionally, we lacked OGTT data to identify previously unknown diabetes in the validation cohort. However, the differences in effect estimates were relatively small when shifting those with newly diagnosed diabetes from the group with diabetes to the group without diabetes in KORA S4 (see Supplementary Table 9, Additional file 2). While validation in the KORA-Age1 cohort strengthens the results for the validated proteins, we may have lacked replication for some proteins particularly if their impact was modified by age since the KORA-Age1 study participants were all older than 65 years. It is worth noting that there is some overlap between the participants in KORA-Age1 and KORA S4 cohort. However, these overlapping participants were examined twice at different time points. Importantly, when we excluded these overlapping participants from our analyses, the results did not show substantial changes. In addition, the shorter follow-up duration in KORA-Age1 (median follow-up time: 6.9 years) compared to KORA S4 (median follow-up time: 15.6 years) should be acknowledged as a limitation in interpreting our findings. To ensure broader applicability, further validation across diverse age groups, ethnicities, and regions are necessary. Moreover, due to the limitations of the GWAS database of incident CHD, the MR analysis performed in this study verified the causal impact of protein biomarkers in the general population rather than in populations with different baseline diabetes status. Additionally, the practical value of the identified proteomic markers for predicting CHD risk needs to be tested in larger studies covering a wider age range.

## Conclusions

In summary, we identified two and 29 validated protein candidates possibly involved in the pathophysiology of CHD among individuals with and without baseline T2D, respectively. Our results provide new insights into a possible causal role of plasma HGF on CHD development and additional support for the involvement of inflammatory processes in CHD development particularly among those without T2D at baseline. Moreover, we established a protein-enriched CHD risk factor-based model which improved the predictive performance of incident CHD in persons with or without T2D compared to the traditional CHD risk factor model. Further research examining larger numbers of T2D patients will be crucial to verify the importance of specific pathways in those with T2D.

### Electronic supplementary material

Below is the link to the electronic supplementary material.

Supplementary Material 1

Supplementary Material 2

## Data Availability

The datasets from this KORA study are not publicly available because the datasets are subject to national data protection laws, and restrictions were imposed by the ethics committee of the Bavarian Chamber of Physicians to ensure data privacy of the study participants. However, datasets are available from the corresponding author on reasonable request through a project agreement from KORA (https://helmholtz-muenchen.managed-otrs.com/external/). Requests should be sent to kora.passt@helmholtz-munich.de and are subject to approval by the KORA board.

## Abbreviations

4E-BP1: Eukaryotic translation initiation factor 4E-binding protein 1
BMI: Body mass index
CD163: Scavenger receptor cysteine-rich type 1 protein M130
cfNRI: Category-free net reclassification index
CHD: Coronary heart disease
C-index: Harrel’s concordance index
CPA1: Carboxypeptidase A1
CVD: Cardiovascular disease
CXCL: C-X-C motif chemokine
EN-RAGE: Protein S100-A12
Ep-CAM: Epithelial cell adhesion molecule
FABP4: Fatty acid-binding protein 4
FDR: False discovery rate
FRS: Framingham Risk Score
FS: Follistatin
GDF-15: Growth differentiation factor 15
GWAS: Available genome-wide association study
HbA1c: Glycated hemoglobin
HB-EGF: Proheparin-binding EGF-like growth factor
HDL-cholesterol: High-density lipoprotein cholesterol
HF: Heart failure
HGF: Hepatocyte growth factor
HOSCAR: Osteoclast-associated immunoglobulin-like receptor
IDI: Integrated discrimination improvement
IL: Interleukin
IL-2RA: Interleukin-2 receptor subunit alpha
IV: Instrumental variable
KIM1: Kidney injury molecule 1
KORA: Cooperative Health Research in the Region of Augsburg
LASSO: Least absolute shrinkage and selection operator
LOD: Limit of detection
ITGB1BP2: Melusin
MI: Myocardial infarction
MMP-12: Matrix metalloproteinase-12
MONICA: Monitoring of Trends and Determinants in Cardiovascular Disease
MR: Mendelian randomization
NT-3: Neurotrophin-3
OGTT: Oral glucose tolerance test
OPN: Osteopontin
OSM: Oncostatin-M
PEA: Proximity extension assay
PGF: Placenta growth factor
T2D: Type 2 diabetes
TGF-alpha: Transforming growth factor-alpha
THBS2: Thrombospondin 2
TNF-R1: Tumor necrosis factor receptor 1
TGM2: Protein-glutamine gamma-glutamyltransferase 2
TNFRSF: Tumor necrosis factor receptor superfamily member
VEGF-A: Vascular endothelial growth factor A
