# Supplementary material for: Association of plasma proteomics with incident coronary heart disease in individuals with and without type 2 diabetes: results from the population-based KORA study
Source: Cardiovasc Diabetol. 2024 Feb 3;23:53. doi: 10.1186/s12933-024-02143-z (PMC10838466; doi:10.1186/s12933-024-02143-z)
Supplement: Supplementary file 1 — Supplementary Material 1 [file 12933_2024_2143_MOESM1_ESM.docx]

**Additional file 1**

**Association of plasma proteomics with incident coronary heart disease in individuals with and without type 2 diabetes: results from the population-based KORA study**

Hong Luo, Marie-Theres Huemer, Agnese Petrera, Stefanie M. Hauck, Wolfgang Rathmann, Christian Herder, Wolfgang Koenig, Annika Hoyer, Annette Peters, Barbara Thorand

**Contents**

[**Supplementary Figure 1** Flow chart of participant exclusions. 2](#_Toc149906120)

[**Supplementary Figure 2** Correlation heatmaps of validated proteins in KORA S4. 3](#_Toc149906122)


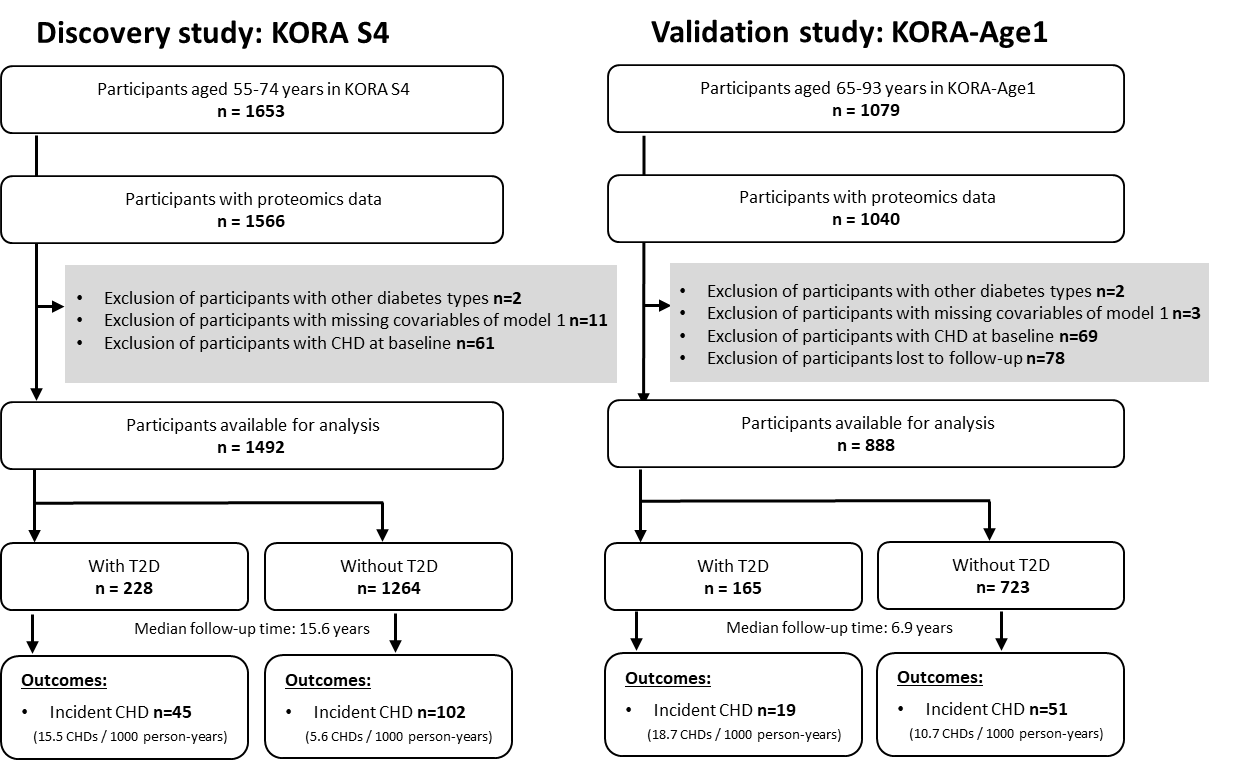


**Supplementary Figure 1** Flow chart of participant exclusions. Model 1 included age, sex, total cholesterol, high‐density lipoprotein cholesterol, systolic blood pressure, antihypertensive medication use, and current smoking. CHD, coronary heart disease; KORA, Cooperative Health Research in the Region of Augsburg; T2D, type 2 diabetes.

**
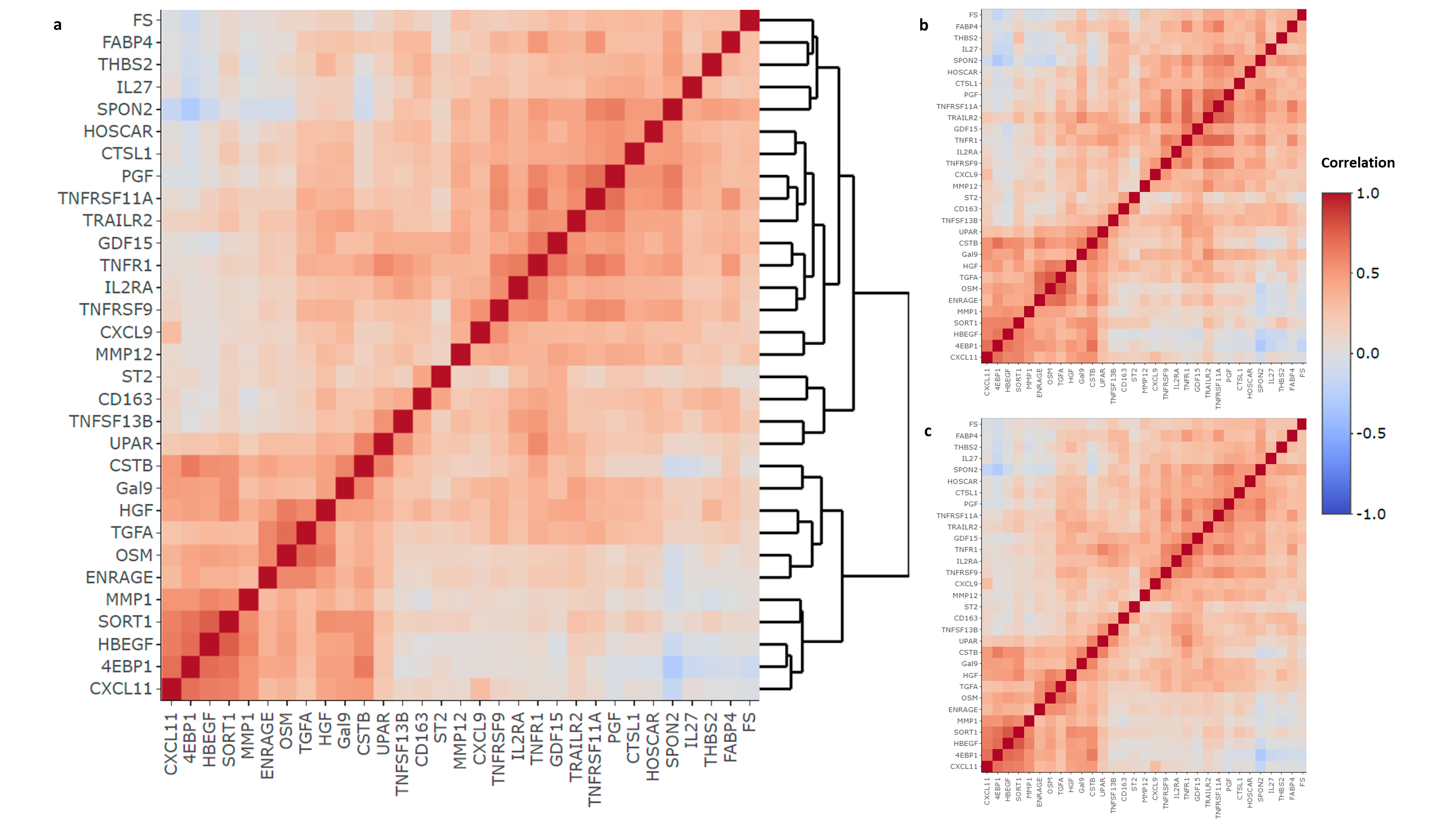
**

**Supplementary Figure 2** Correlation heatmaps of validated proteins in KORA S4. Pearson correlation heatmap of the proteins with the corresponding hierarchical clustering dendrogram is shown in **a**. among the general population; **b**. among individuals with baseline type 2 diabetes and **c**. among individuals without baseline type 2 diabetes (without hierarchical clustering to fix the order of markers).
